# Comparison of capillary zone and agarose gel protein electrophoresis in macaws (*Ara* spp.)

**DOI:** 10.3389/fvets.2026.1902929

**Published:** 2026-09-18

**Authors:** Jared S. Zion, Anthony A. Pilny, Stephanie K. Lamb, Fabiano Montiani-Ferreira, Carolyn Cray

**Affiliations:** 1 Arizona Exotic Animal Hospital, Phoenix, AZ, United States; 2 Department of Pathology and Laboratory Medicine, Division of Comparative Pathology, University of Miami Miller School of Medicine, Miami, FL, United States; 3 Departamento de Medicina Veterinária, Universidade Federal do Paraná, Curitiba, Brazil

**Keywords:** agarose gel, avian, capillary zone, macaw, prealbumin, protein electrophoresis, psittacine

## Abstract

The avian inflammatory response is complex and requires integration of clinical examination findings alongside hematologic, biochemical, and protein electrophoretic data to best characterize the acute phase and immune response. Interpretation of protein electrophoresis in birds, however, is challenging due to methodological variability, inconsistent fraction resolution, and the lack of established species- and method-specific reference intervals. Psittacine birds represent the largest family of companion avian patients in clinical practice, emphasizing the need for reliable and clinically applicable data for diagnostic interpretation. This study compared agarose gel electrophoresis (AGE) and capillary zone electrophoresis (CZE) in 42 client-owned macaws (*Ara* spp.) complemented by paired serum-plasma analysis. Birds were classified as clinically normal or abnormal based on examination findings and clinicopathologic data. Capillary zone electrophoresis resolved seven protein fractions compared to six fractions resolved by AGE. Method comparison statistics demonstrated weak to moderate correlation, but poor agreement by Bland–Altman analysis, with systematic bias across all fractions, confirming results between methods are not equivalent. In an exploratory investigation, the abnormal group was found to demonstrate significantly increased total leukocyte and heterophil counts, with negative correlations between these parameters and the albumin-to-globulin ratio. Notable electrophoretic alterations were observed in select diseased birds. Agarose gel electrophoresis and CZE yielded analytically different results in the samples. These findings support the need for method-specific reference intervals and emphasize the importance of integrating protein electrophoresis results with clinical and hematologic findings.

## Introduction

Protein electrophoresis (EPH) remains a valuable diagnostic tool in avian medicine for characterization of the acute phase and immune responses and for quantification of albumin compared to bromocresol green dye-binding methods (1–6). Agarose gel electrophoresis (AGE) is one method of EPH commonly used in veterinary diagnostic laboratories; however, studies in humans and various animal species have reported that capillary zone electrophoresis (CZE) may provide improved fraction resolution, faster analytic time, and reduced sample handling compared to AGE (6–9). Interpretation of avian electrophoretic fractions remains challenging because the composition of protein fractions is incompletely characterized, and fraction migration may vary substantially among avian taxa and underlying health status (4).

Psittacine birds represent the most common companion bird group presenting to veterinary hospitals (10). Characteristic electrophoretic profiles in birds have been associated with several infectious diseases such as chlamydiosis, mycobacteriosis, avian bornavirus, and aspergillosis (11–15). Electrophoresis interpretation in birds can be complicated by non-pathologic factors including reproductive status, seasonal variation, and sex-related differences as well as subclinical disease (4, 16–19). In a retrospective review of psittacine birds with reported illness, abnormalities identified on EPH did not consistently overlap with complete blood count (CBC) abnormalities, suggesting the complementary diagnostic value of these modalities (19).

Comparative studies between AGE and CZE methodologies in non-psittacine birds have repeatedly demonstrated significant differences in protein fraction quantification, emphasizing the need for method-specific and species-specific reference intervals (8, 20, 21). To date, no studies have directly compared AGE and CZE methods in a psittacine bird species. The present study aimed to analyze electrophoretic profiles generated by AGE and CZE in macaws (*Ara* spp.) and to explore the clinical implications between electrophoresis results and hematologic indicators of disease. In addition, preliminary method-specific reference intervals using paired serum-plasma samples for macaws were calculated.

## Materials and methods

Forty-two client-owned macaws presenting for voluntary veterinary evaluation were included in the study and consisted of the following species: Blue & Gold (*n* = 13), Green Winged (*n* = 8), Red-fronted (*n* = 4), Severe (*n* = 3), Hyacinth (*n* = 2), Military (*n* = 2), Miligold (*n* = 2), Catalina (*n* = 2), Blue-throated (*n* = 1), Scarlet (*n* = 1), Ruby (*n* = 1), Harlequin (*n* = 1), Flame (*n* = 1), and an unknown hybrid (*n* = 1). Written, informed consent of participation in the study was obtained from each owner. Each macaw underwent a physical examination including hands-off observation, comprehensive palpation, and cardio-pulmonary auscultation. A detailed clinical history including documentation of any presenting concerns, medical history, diet, and egg-laying history, if apparent, were recorded.

Approximately 2 mL of blood was collected from each macaw using a 25-gauge needle from the right jugular vein or medial metatarsal vein. Samples were distributed into EDTA microtainers (BD Microtainer tube, Franklin Lakes, NJ, USA) for CBC and lithium heparin microtainers for plasma biochemistry and EPH. Blood was also distributed into serum separator microtainers for paired plasma-serum analysis from 27 patients. Heparinized blood was centrifuged at 3000 revolutions per minute for 10 min and plasma decanted into sterile tubes. The same procedure was performed with serum samples after allowing blood to clot for approximately 7 min. Blood smears were made immediately after collection without anticoagulant, kept at room temperature, and submitted to the diagnostic laboratory with the blood tubes for evaluation. Blood tubes were kept under refrigeration prior to submission, and an icepack was placed in the shipping container of each respective sample when submitted within 24–72 h of collection. The individuals performing fraction delimits for the electrophoresis were blinded to the health status of the patient.

Birds were classified as clinically normal (*n* = 24) or clinically abnormal (*n* = 18) based on owner-reported clinical signs, physical examination findings, and/or clinicopathologic abnormalities considered clinically relevant at the time of evaluation. Birds were considered clinically normal if physical examination did not support systemic disease, the client did not report concerns, and hematologic and biochemical evaluations were within normal limits. Common clinical presentations among clinically abnormal birds included gastrointestinal, respiratory, musculoskeletal/traumatic and reproductive-related conditions. Birds with multiple concurrent abnormalities, including clinically relevant hematologic abnormalities such as anemia or severe leukocytosis, were categorized within the overall clinically abnormal group based on their respective reported concerns, physical examination findings, and clinicopathologic abnormalities.

Biological sex was confirmed in all birds. Whole blood samples from 25 birds were submitted for sex determination DNA testing (University of Georgia Infectious Disease Laboratory, Athens, GA 31793 USA). Birds that did not undergo DNA testing had provided previous documentation of sex-confirmed testing or had an egg-laying history.

Routine hematology and biochemistry were performed at the Avian & Wildlife Laboratory (University of Miami, Miami, FL 33136 USA). The hemacytometer method using phloxine dye was utilized in tandem with WBC differential counts performed on smears at 1000x. White blood cell and RBC morphology were also evaluated. Biochemistry testing was performed on a Vitros 5,600 analyzer (Quidel-Ortho, San Diego, CA 92121 USA) and included the following analytes: glucose, BUN, creatinine, sodium, potassium, chloride, CO2, amylase, lipase, calcium, phosphorus, magnesium, cholesterol, uric acid, triglycerides, total protein, AST, CPK, GGT, bile acids, and GLDH.

### Agarose gel electrophoresis

Samples were analyzed using split beta gels and the SPIFE 3000 system (Helena Laboratories, Inc., Beaumont, TX, 77707 USA). The albumin/globulin (A/G) ratio was calculated by dividing the sum of prealbumin and albumin by the sum of globulin fractions. The equipment was maintained according to manufacturer instructions. Normal and abnormal human control samples (Helena Laboratories, Inc.) were also included in the gels. Total protein was determined by using the biuret method and a chemistry analyzer (Ortho Integrated Systems Vitros 5,600; Quidel-Ortho, San Diego, CA, 92121 USA). Intra-assay coefficient of variation (CV) analysis was conducted examining four samples analyzed six times within one run. The mean values were as follows: prealbumin - 4.0%, albumin–1.9%, alpha 1–9.8%, alpha 2–6.8%, beta–4.0%, gamma–7.1%. Inter-assay CV was conducted examining four samples analyzed over five days. The mean values were as follows: prealbumin–7.6%, albumin–2.4%, alpha 1–10.1%, alpha 2–6.0%, beta–2.3%, gamma–10.1%.

### Capillary zone electrophoresis

Samples were analyzed by manufacturer protocols for Urine Protein Electrophoresis (Sebia Capillarys 2 Flex Piercing, Norcross, GA 30092 USA). Samples were diluted 1:8 using urine running buffer as diluent. This buffer has been observed to aid in fraction migration and resolution of avian proteins by placing anodic and cathodic stop points (20). Absolute values for each fraction were obtained by multiplying the percentages by the total protein concentration as determined using biuret method. The A/G ratio was calculated by dividing the sum of prealbumin and albumin by the sum of globulins fractions. Human control samples were used. Intra-assay CV analysis was conducted examining four samples analyzed six times within one run. The mean values were as follows: prealbumin - 0.8%, albumin - 0.5%, alpha 1–5.1%, alpha 2–3.9%, beta–2.5%, gamma 1–3.5%, gamma 2–7.2%. Inter-assay CV analysis was conducted examining four samples over five days. The mean values were as follows: prealbumin–2.7%, albumin–1.2%, alpha 1–5.4%, alpha 2–5.1%, beta- 5.2%, gamma 1–3.8%, gamma 2–5.0%.

### Statistical analysis

All statistical analyses were performed using MedCalc Statistical Software version 20.027 (MedCalc Software Ltd., Ostend, 8,400 Belgium) and Python 3.11 (Python Software Foundation, Wilmington, DE, 97008 USA). Statistical significance was set at *p* < 0.05. Distribution was assessed using the D’Agostino–Pearson omnibus normality test (22). For reference interval (RI) determination, a threshold of *p* < 0.30 was used to classify measurands as non-Gaussian in accordance with ASVCP recommendations for veterinary reference interval determination (23). Measurands demonstrating Gaussian distributions were analyzed using the parametric method, whereas measurands with non-Gaussian distributions were analyzed using the robust method. For group comparisons and correlations, the conventional threshold of *p* < 0.05 was applied. Outliers and suspect data were evaluated using Tukey’s method; no data points were excluded. Data are presented as mean ± standard deviation (SD) for Gaussian variables and median with interquartile range (IQR) for non-Gaussian variables. Sample size was determined *a priori* based on ASVCP recommendations and conventional power analysis methodology. For reference interval generation, sample size was determined in accordance with the ASVCP Reference Interval Guidelines (23). These guidelines recommend inclusion of at least 20 clinically normal individuals for preliminary reference interval generation. Accordingly, enrollment of 24 clinically normal macaws slightly exceeded this minimum recommendation; however, the reference intervals generated in the present study should be considered preliminary and warrant validation in larger populations. For the remaining primary analytical objectives, sample size requirements were estimated using formal statistical power analyses. For group comparisons (24 clinically normal vs. 18 clinically abnormal birds, total *n* = 42), standard two-sample power calculations based on Cohen’s standardized effect size indicated that a minimum of approximately 34 total subjects (17 per group) would provide 80% power to detect a moderate-to-large effect size (*d* = 0.80) at *α* = 0.05 (two-tailed). For correlation analyses (*n* = 42), power calculations based on Fisher’s z-transformation indicated that approximately 37 subjects would provide 80% power to detect a moderate correlation (*r* = 0.40) at *α* = 0.05. Accordingly, the study met minimum sample size requirements for detecting moderate-to-large effects in its primary analytical objectives involving preliminary reference interval generation. Sex-based differences were explored descriptively from the clinically normal macaws.

Paired comparisons between plasma and serum samples (*n* = 27) were performed using paired t-tests for Gaussian paired differences and Wilcoxon signed-rank tests for non-Gaussian paired differences. Mean paired differences (plasma − serum) were calculated and reported. No adjustment for multiple comparisons was applied for predefined analytical evaluations. Paired serum and plasma samples were compared to assist in delineation of the beta-globulin fraction, as fibrinogen, present in plasma, migrates within the beta-globulin region on AGE in avian species (4).

Comparisons of data from clinically normal and abnormal birds were performed. Group comparisons were conducted using Welch’s *t*-test for Gaussian variables and Mann–Whitney *U*-test for non-Gaussian variables. To account for multiple comparisons across electrophoretic fractions, Holm–Bonferroni correction was applied. Both unadjusted and adjusted *p*-values are reported. Effect sizes were calculated using Cohen’s d for parametric comparisons and Cliff’s delta for non-parametric comparisons. Associations between CBC parameters and CZE fractions (albumin g/dL, total gamma g/dL, and A/G ratio) were evaluated using Spearman’s rank correlation coefficient (rs). Correlation strength was interpreted as: very strong (>0.70), strong (0.40–0.69), moderate (0.30–0.39), weak (0.20–0.29), and negligible (<0.20). Method comparison between CZE and AGE included Pearson’s or Spearman’s correlation analysis depending on distribution; Passing–Bablok regression to assess constant and proportional bias; Cusum test for linearity; and Bland–Altman analysis to evaluate agreement and systematic bias (24). Passing–Bablok regression was interpreted as evidence of constant error when the 95% CI of the intercept did not include 0, and proportional error when the 95% CI of the slope did not include 1.

## Results

Birds in the study ranged in age from 6 months to 41 years. The median age of the clinically normal bird group was 15 years with an interquartile range (IQR) of 17 years, compared with a median age of 26.5 years and an IQR of 21.5 years in the clinically abnormal bird group. The normal group consisted of 15 males and 9 females, whereas the abnormal group included 10 males and 8 females.

### Fraction resolution and delimitation

Seven fractions consisting of prealbumin, albumin, alpha 1, alpha 2, beta, gamma 1, and gamma 2 globulins were resolved by CZE (Figure 1). For AGE, prealbumin, albumin, alpha 1, alpha 2, beta, and a single gamma fraction were quantified. The mean beta-globulin fraction in CZE was greater in plasma (9.7% ± 3.0) than in serum (5.0% ± 1.4). This difference was statistically significant (*p* < 0.001). Preliminary reference intervals for CZE and AGE were calculated from the clinically normal population (Tables 1, 2).

**Figure 1:**
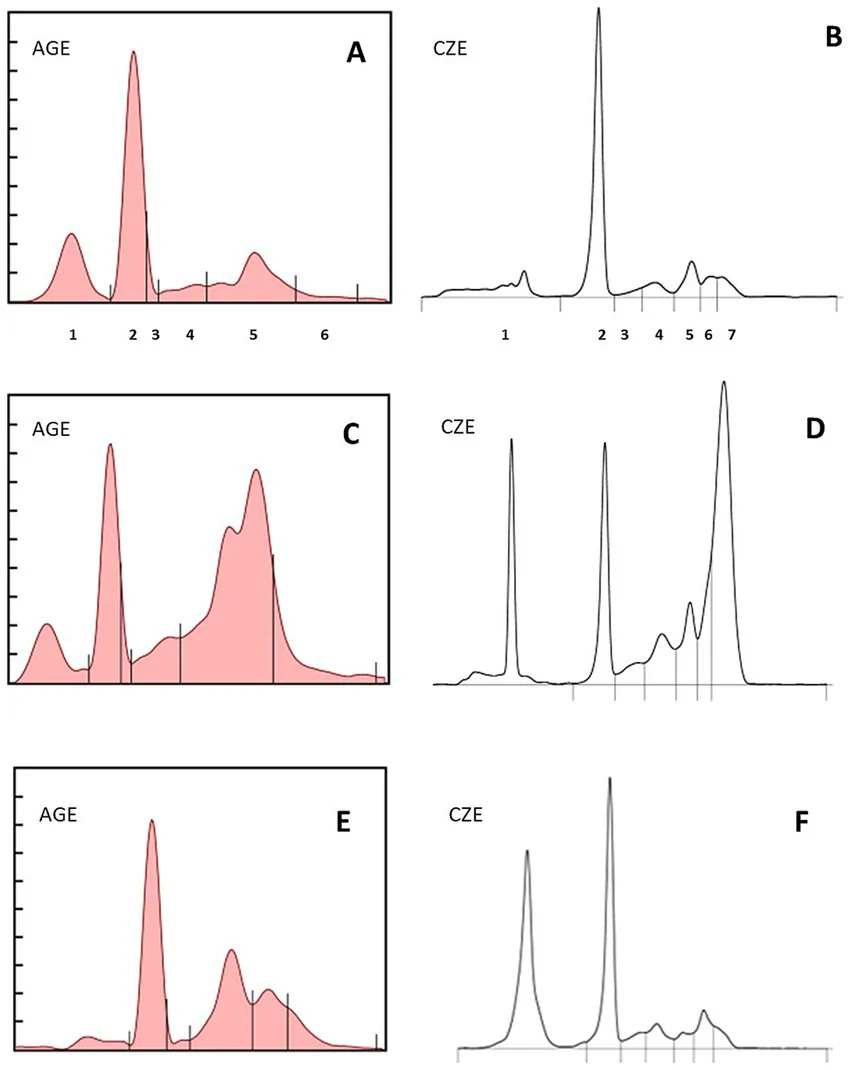
Representative paired AGE and CZE electrophoretograms from a 13-year-old clinically normal, male Blue and Gold **(A,B)**, with A/G ratio 2.05 and 2.38, respectively. For AGE, the fractions are as follows: 1, prealbumin, 2, albumin, 3, alpha 1, 4, alpha 2, 5, beta, and 6, gamma. For CZE, the fractions are as follows: 1, prealbumin, 2, albumin, 3, alpha 1, 4, alpha 2, 5, beta, 6, gamma 1, 7, gamma 2. Panels **(C,D)** were obtained from an 11-year-old male Severe with confirmed avian bornaviral infection and clinical proventricular dilatation disease. The A/G ratio was 0.50 (AGE) and 0.47 (CZE). Note in **C** (AGE), the pronounced beta globulin fraction whereas **D** (CZE), shows a defined prealbumin fraction and elevation in gamma 2 globulins. Panels **(E,F)** were obtained from a 25-year-old female Blue and Gold with an inappropriate and excessive egg-laying history; sample collected 48 h prior to laying. The A/G ratio was 0.80 (AGE) and 2.98 (CZE). Note the increased alpha 2 globulin fraction in **E** (AGE) vs. the pronounced prealbumin fraction in **F** (CZE).

**Table 1:** Reference intervals for plasma protein fractions determined by CZE (*n* = 24).

Measurand	Mean (SD)	Median	Min-Max	*p*-value	Dist./Method	LRL (90% CI)	URL (90% CI)
Total protein, g/dL	3.7 (0.8)	3.7	2.2–6.0	0.04	NG/R	2.3 (1.7–3.0)	5.0 (4.4–5.7)
A/G Ratio	2.09 (0.46)	1.99	1.26–3.15	0.30	NG/R	1.13 (0.91–1.44)	2.89 (2.45–3.27)
Prealbumin, g/dL	0.48 (0.30)	0.40	0.26–1.73	<0.0001	NG/R	0.11 (0.05–0.20)	0.70 (0.54–0.83)
Prealbumin, %	12.4 (4.6)	11.7	6.4–28.8	<0.0001	NG/R	4.9 (2.3–7.0)	18.6 (16.2–22.0)
Albumin, g/dL	2.03 (0.38)	2.01	1.18–2.83	0.83	G/P	1.28 (1.08–1.52)	2.78 (2.53–2.97)
Albumin, %	54.5 (4.1)	55.2	46.6–60.6	0.46	G/P	46.5 (44.4–49.6)	62.5 (60.7–63.8)
Alpha 1, g/dL	0.11 (0.03)	0.11	0.07–0.16	0.56	G/P	0.06 (0.05–0.07)	0.16 (0.18–0.18)
Alpha 1, %	3.0 (0.6)	3.0	1.9–4.3	0.48	G/P	1.8 (1.6–2.1)	4.2 (3.8–4.6)
Alpha 2, g/dL	0.26 (0.06)	0.25	0.17–0.41	0.04	NG/R	0.15 (0.11–0.18)	0.35 (0.31–0.40)
Alpha 2, %	7.0 (1.2)	6.8	5.2–9.5	0.40	G/P	4.7 (4.3–5.3)	9.3 (8.5–10.0)
Beta, g/dL	0.37 (0.11)	0.36	0.13–0.59	0.97	G/P	0.15 (0.09–0.22)	0.59 (0.51–0.64)
Beta, %	9.8 (2.3)	10.3	5.1–13.5	0.33	G/P	5.3 (4.0–7.0)	14.3 (13.2–15.1)
Gamma 1, g/dL	0.24 (0.07)	0.21	0.12–0.44	0.04	NG/R	0.08 (0.05–0.13)	0.36 (0.29–0.42)
Gamma 1, %	6.3 (1.4)	6.2	4.1–8.9	0.64	G/P	3.5 (3.0–4.3)	9.1 (8.2–9.7)
Gamma 2, g/dL	0.26 (0.13)	0.20	0.11–0.62	0.03	NG/R	0.03 (0.0–0.06)	0.46 (0.30–0.59)
Gamma 2, %	6.8 (3.1)	5.8	3.4–14.1	0.06	NG/R	0.4 (0–2.9)	11.1 (7.5–14.9)

NG, Non Gaussian; G, Gaussian; R, Robust method; P, Parametric Method.

**Table 2:** Reference intervals for plasma protein fractions determined by AGE (*n* = 24).

Measurand	Mean (SD)	Median	Min–Max	*p*-value	Dist./Method	LRL (90% CI)	URL (90% CI)
Total protein, g/dL	3.7 (0.8)	3.7	2.2–6.0	0.04	NG/R	2.3 (1.7–3.0)	5.0 (4.4–5.7)
A/G Ratio	1.99 (0.48)	1.92	0.85–2.77	0.83	G/P	1.06 (0.79–1.36)	2.93 (2.65–3.15)
Prealbumin, g/dL	0.60 (0.17)	0.59	0.30–1.14	0.001	NG/R	0.31 (0.20–0.42)	0.85 (0.75–0.97)
Prealbumin, %	16.4 (3.4)	16.6	9.5–22.4	0.68	G/P	9.7 (7.9–12.0)	23.1 (21.4–24.4)
Albumin, g/dL	1.82 (0.43)	1.83	1.01–3.00	0.15	NG/R	1.02 (0.73–1.32)	2.59 (2.32–2.91)
Albumin, %	49.4 (4.9)	49.7	36.2–59.0	0.33	G/P	39.7 (36.4–43.1)	59.0 (55.7–61.6)
Alpha 1, g/dL	0.10 (0.04)	0.09	0.04–0.18	0.33	G/P	0.02 (0.0–0.04)	0.18 (0.15–0.20)
Alpha 1, %	2.6 (1.0)	2.3	1.5–5.5	0.03	NG/R	0.5 (0.1–1.1)	4.4 (3.2–5.2)
Alpha 2, g/dL	0.27 (0.06)	0.26	0.18–0.42	0.10	NG/R	0.16 (0.13–0.19)	0.36 (0.32–0.41)
Alpha 2, %	7.4 (1.7)	7.4	4.3–10.8	0.68	G/P	4.1 (3.4–5.1)	10.8 (9.8–11.5)
Beta, g/dL	0.63 (0.20)	0.60	0.28–1.02	0.79	G/P	0.24 (0.15–0.34)	1.03 (0.89–1.14)
Beta, %	17.2 (4.4)	16.3	9.1–30.6	0.01	NG/R	8.9 (6.3–11.5)	24.0 (20.7–27.7)
Gamma, g/dL	0.26 (0.11)	0.24	0.10–0.57	0.03	NG/R	0.04 (0.04–0.12)	0.44 (0.35–0.54)
Gamma, %	7.1 (2.4)	7.4	3.1–13.0	0.70	G/P	2.3 (1.3–3.6)	11.8 (10.2–13.2)

Note that AGE resolves only one gamma globulin fraction, noted as Gamma here. NG, non gaussian; G, gaussian; R, robust method; P, parametric method.

### Comparison of AGE and CZE

The results of the three statistical methods of comparative analysis are illustrated in Table 3.

**Table 3:** Passing–Bablok regression, Bland–Altman analysis, and Spearman correlation of fractions quantitated by AGE and CZE (*n* = 42).

Fraction	AGE Median (IQR)	CZE Median (IQR)	Correlation *r*-value (*p*-value)	Passing–Bablok *y*-intercept [95% CI]	Passing–Bablok slope [95% CI]	Bland–Altman bias Mean (SD)
Albumin-to-globulin ratio	1.90 (0.50)	1.97 (0.58)	0.54 (0.0003)	0.07 [−0.70, 0.61]	1.00 [0.69, 1.44]	0.11 (0.54)
Prealbumin (%)	16.50 (5.30)	12.30 (5.82)	0.32 (0.04)	−8.80 [−26.89, −0.26]	1.30 [0.79, 2.31]	−2.54 (7.61)
Albumin (%)	47.60 (5.75)	53.90 (7.60)	0.53 (0.0003)	−3.45 [−24.92, 18.33]	1.16 [0.72, 1.61]	4.03 (6.28)
Alpha 1 globulin (%)	2.35 (1.38)	3.15 (1.15)	−0.18 (0.25)	1.39 [−1.30, 2.42]	0.75 [0.25, 2.00]	0.78 (1.49)
Alpha 2 globulin (%)	7.20 (2.80)	6.65 (1.62)	0.23 (0.14)	2.78 [−0.18, 5.23]	0.53 [0.19, 0.96]	−1.03 (4.02)
Beta globulin (%)	16.65 (4.62)	10.25 (3.45)	0.26 (0.09)	−2.02 [−8.99, 4.94]	0.70 [0.30, 1.13]	−8.10 (6.70)
Gamma globulin (%)	7.55 (2.95)	13.35 (4.48)	0.31 (0.05)	−1.85 [−11.99, 3.33]	2.00 [1.30, 3.43]	6.83 (6.32)

Note that the gamma globulin comparison was between total gamma globulins (gamma 1 + gamma 2) by CZE versus the gamma globulin fraction (AGE).

AGE, agarose gel electrophoresis; A/G, albumin/globulin; CZE, capillary zone electrophoresis; IQR, interquartile range; CI, confidence interval; r, correlation coefficient. Normality was evaluated using the D’Agostino–Pearson test.

Spearman’s rank correlation analysis indicated significant correlation between methods, ranging from weak to moderate across prealbumin, albumin, and total gamma globulins, as well as the A/G ratio. No significant correlation was observed for alpha 1, alpha 2, or beta globulins. Passing–Bablok regression demonstrated systematic deviation from identity for three fractions. Constant error was observed in prealbumin and proportional error was observed in alpha 2 and gamma globulins. Bland–Altman analysis confirmed systematic bias across all fractions. Mean differences (CZE − AGE) ranged from −8.10% (beta globulin) to +6.83% (gamma globulin). Wide limits of agreement were observed particularly for prealbumin, beta globulin, and gamma globulin. The difference between the electrophoretic methods for each fraction is further illustrated by Bland–Altman plots (Figure 2).

**Figure 2:**
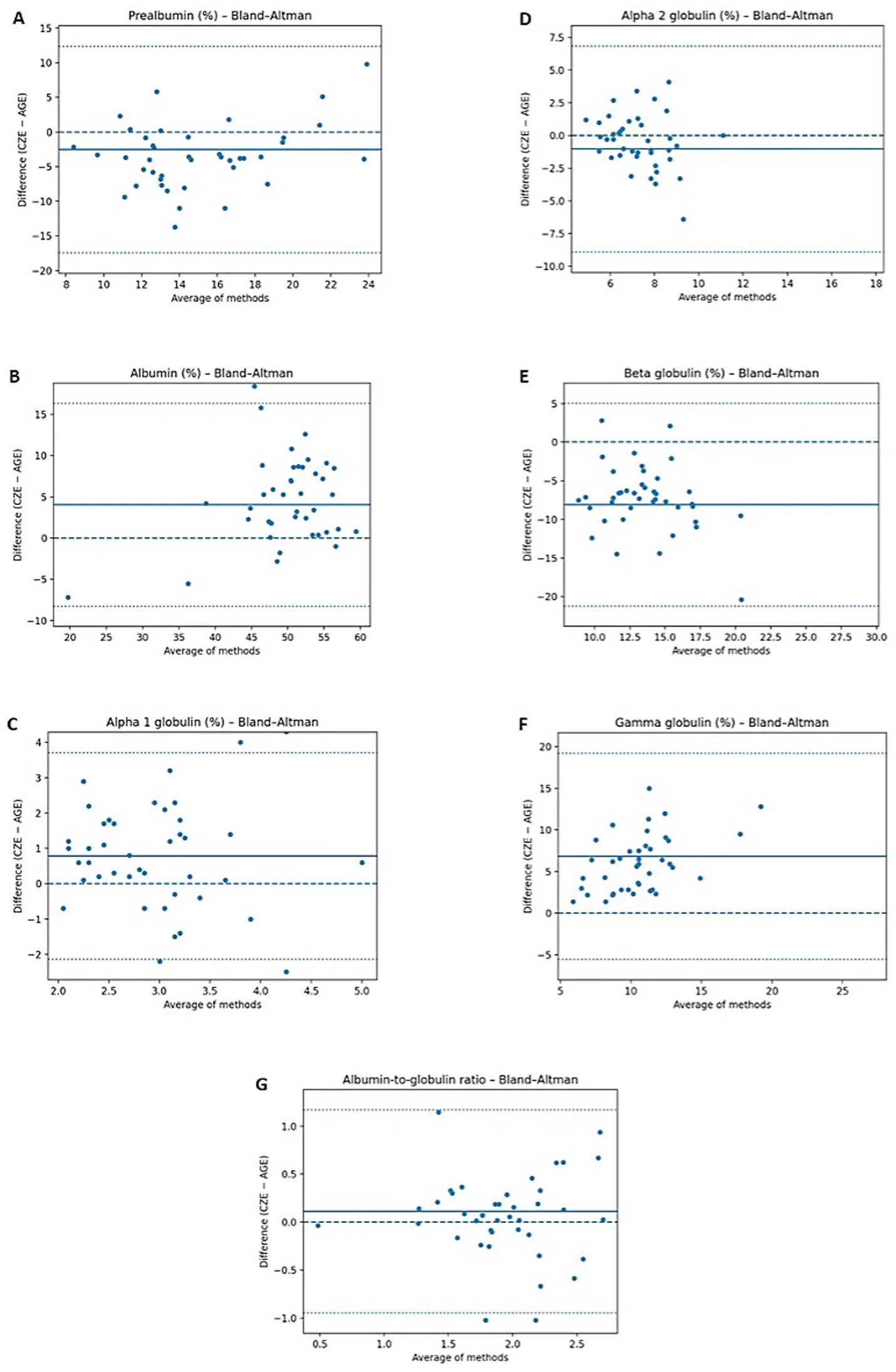
Bland–Altman for electrophoresis fractions in macaws plotted as the difference between the methods (CZE–AGE) on the *y*-axis and the average of the methods on the *x*-axis. The dashed blue line represents the 95% limits of agreement, and the dotted blue line indicates the mean difference between the two methods. **(A)** Prealbumin, **(B)** albumin, **(C)** alpha 1 globulin, **(D)** alpha 2 globulin, **(E)** beta globulin, **(F)** gamma globulin, **(G)** albumin-to-globulin ratio.

### Clinically normal vs. abnormal macaws

No statistically significant differences were present between CZE fractions of clinically normal and abnormal macaws (*p* > 0.05). However, differences in total WBC (*p* = 0.04), total heterophils (*p* = 0.02), and total lymphocytes (*p* = 0.03) but not PCV (*p* = 0.20) were observed. Using Spearman’s rank correlation analysis, a significant negative correlation was observed between total leukocyte count and A/G ratio (rs = −0.42, *p* = 0.007). A similar relationship was observed between absolute heterophil count and A/G ratio (rs = −0.44, *p* = 0.004). Of the 18 samples from clinically abnormal birds, the most common electrophoretic change was a mild to moderate increase in gamma 2 globulins (14/18) but there were no consistent changes across the other fractions.

Select electrophoretogram comparisons of AGE and CZE of individuals in the study, alongside their health status and associated clinical diagnosis, are provided to illustrate distinct differences observed between the methods (Figure 1). One bird with confirmed avian bornaviral infection and clinical proventricular dilatation disease demonstrated marked expansion of the prealbumin (CZE:15.7%) and gamma (CZE gamma 2: 38.7%) fractions, as well as a moderate decrease in albumin percentage on CZE compared with AGE (Figures 1C,D). In an inappropriately reproductively active female macaw with concurrent severe leukocytosis (46.9 × 10^3^ cells/μL), differing A/G ratios between AGE and CZE (0.80 and 2.98, respectively) and a prominent prealbumin fraction (CZE: 41.4%) were observed (Figures 1E,F).

### Sex based differences

Due to limited subgroup sample sizes in the normal group (males *n* = 15; females *n* = 9), inferential statistical testing was not considered reliable to be performed, as the study was underpowered to detect small-to-moderate effect sizes. Therefore, results are presented descriptively to identify potential trends. To provide an estimate of the magnitude of differences between groups, standardized effect sizes (Cohen’s *d*) were calculated. Mean values with standard deviation for albumin and alpha fractions were comparable between sexes, with small effect sizes observed for albumin (CZE % albumin: 55.1 ± 3.9 in males vs. 53.7 ± 4.3 in females; *d* = 0.33; AGE % albumin: 50.1 ± 5.4 vs. 48. ± 3.9; *d* = 0.42). Alpha fractions were likewise similar, with CZE % alpha 1 (2.9 ± 0.6 vs. 3.2 ± 0.5; *d* = 0.53) and CZE % alpha 2 (7.2 ± 1.4 vs. 6. ± 0.6; *d* = 0.49) showing overlapping distributions and small-to-moderate effect sizes. A trend toward higher gamma globulin fractions was observed in females compared to males in both methods, with CZE % total gamma of 12.5 ± 3.5 in males vs. 14.2 ± 3.7 in females (*d* = 0.47), and AGE % gamma of 6.4 ± 2.2 vs. 8.1 ± 2.6 (*d* = 0.73); however, substantial overlap between groups was present.

## Discussion

This study represents the first comparison of AGE and CZE methodologies in a psittacine bird species and demonstrates that analytical differences exist between methods. Similar to comparative studies in other birds (8, 20, 21), CZE identified additional and more discrete fractions compared to AGE; however, agreement between methods was inconsistent across multiple fractions, including prealbumin, albumin, and gamma globulins. Systematic bias was identified across all fractions by Bland–Altman analysis. Passing–Bablok regression demonstrated constant error in prealbumin and proportional error in alpha 2 and gamma globulins, while correlations between methods ranged from weak to moderate for several fractions (Figure 2; Table 3). These findings indicate that AGE and CZE should not be considered analytically equivalent methods for macaw species.

The improved fraction resolution on CZE relative to AGE has been well observed in multiple non-psittacine birds (8, 20, 21). Migration characteristics of electrophoretic fractions have been observed to differ among avian species and between electrophoretic methods, illustrating that direct comparison between techniques must be interpreted cautiously (4, 8). In black kites and pigeons, proteins migrating in the alpha fractions on AGE were noted to shift in the prealbumin region on CZE (8). This degree of migration was not observed in the macaws, yet differing A/G ratio ranges and significant method-dependent differences were observed between the two methods (Tables 1–3). In a study involving bald eagles using identical electrophoretic methodologies to those used in the present study, significant correlations between CZE and AGE were identified for the prealbumin fraction, gamma globulin fraction and A/G ratio (20). Unlike the bald eagle study, albumin fractions were also significantly correlated between AGE and CZE in macaws. The clinical significance of these interspecies analytical differences remains unclear. These findings further support that avian electrophoretic patterns are species-dependent and that direct extrapolation of reference intervals across the Aves class is not appropriate. In addition to the identification of proteins that migrate in these fractions, the clinical interpretation of individual fractions requires knowledge of the underlying health status, which we aimed to illustrate through evaluation of clinically normal and abnormal macaws.

Through hematologic evaluation, clinically abnormal birds demonstrated significant leukocyte alterations, including increased total white blood cell, heterophil, and lymphocyte counts. Higher total white blood cell and heterophil counts were associated with lower A/G ratios. In contrast, the electrophoretic fraction comparison between clinically normal and abnormal groups did not reach statistical significance. The relatively small sample size and the heterogenous disease processes represented by the abnormal group, combined with a study design powered to detect moderate-large effects (*d* = 0.80), may have reduced statistical power to detect disease-associated or disease-specific electrophoretic patterns. This is further showcased by 78% of birds in the abnormal group demonstrating increased gamma-2 globulins despite a lack of group-level significance, further suggesting that a Type II (false-negative) error cannot be completely excluded. Additional studies involving larger cohorts and more homogenous disease conditions are warranted to better characterize electrophoretic alterations associated with specific inflammatory and infectious diseases in macaws. The inflammatory-associated changes reflected by the A/G ratio may accompany leukocyte abnormalities and reinforce the complementary value of integrating CBC with EPH results in the assessment of inflammatory and infectious diseases in macaws. Prospective studies evaluating specific acute phase proteins alongside these diagnostic tools may further prove valuable due to the migration characteristics of acute phase proteins in birds.

Despite a lack of statistically significant electrophoretic differences between clinically normal and abnormal groups, select cases revealed notable electrophoretic observations. One representative case with confirmed avian bornaviral infection and clinical proventricular dilatation disease showed an expanded and visually distinct gamma-globulin region on CZE compared to AGE (Figures 1C,D). Previous studies in African Grey parrots with experimentally-induced bornaviral infection demonstrated significant gamma-globulin elevations with AGE (14). The visually distinct protein alterations in this case could support that the increased fraction resolution provided by CZE may improve characterization of electrophoretic alterations associated with inflammatory responses in psittacine birds. While it is considered rare that protein electrophoresis is solely diagnostic for any one disease, perhaps with the exception of lymphoproliferative disease (i.e., presence of a monoclonal band), CZE does provide higher resolution and reproducibility, such that larger focused studies could examine electrophoretic changes in response to particular stimuli or disease over time to evaluate if particular patterns or protein changes evolve. These types of applications may be situational, as reported previously with the use of AGE in penguins with aspergillosis, but should be investigated further (4).

Several acute phase proteins including serum amyloid A, ovotransferrin, and other globulin-associated proteins have been identified in multiple avian species with inflammatory diseases (25–30). Fibrinogen has been associated with increased beta-globulin region migration in plasma AGE across several avian species (4, 16) and a shift toward the gamma-globulin region in the Common Mynah when using cellulose acetate electrophoresis (5). In the present study, significant differences in the beta-globulin region were observed between plasma and serum samples on CZE, likely reflecting, at least in part, fibrinogen-associated migration. This difference is clinically relevant when EPH is performed, illustrating the importance of sample type (i.e., serum vs. plasma) prior to interpretation.

The avian prealbumin fraction remains poorly characterized, with variability in resolution, migration and quantification reported across species and electrophoretic methodologies (4, 20, 21, 31–33). In the present study, the prealbumin fraction was identified across both AGE and CZE. Biologic factors, including sex and hormonal activity, may also contribute to the prealbumin fraction in birds (4, 34). Sex determination testing was pursued to evaluate potential sex-associated differences within the clinically normal population; however, statistical inference was limited by subgroup sample size. Significantly increased prealbumin fractions have been reported in actively laying hens compared to non-laying hens on CZE (35). Similarly, the electrophoretogram illustrated in Figures 1E,F was obtained from a macaw with a chronic, inappropriate egg-laying history that produced an egg 48 h following sample collection. These observations may suggest that oviposition and reproductive activity may influence prealbumin-associated patterns in psittacine birds and support the premise that reproductive status should be considered when interpreting EPH in birds. Further studies evaluating the effects of sex and reproductive status on the avian electrophoretogram using larger populations are needed.

The preliminary reference intervals calculated in this study should further be interpreted amid the context of potential interspecies variation within the genus *Ara*. The macaw population involving several species precluded meaningful evaluation of species-specific differences within this genus. Interspecies and physiologic differences evaluated by electrophoretic profiles among psittacine birds have yet to be definitively established, however, future studies evaluating individual macaw species may further evaluate this potential effect. Additionally, advanced screening for subclinical disease beyond baseline bloodwork was not performed in the clinically normal group. Diet, housing, and fasting status, were not systematically evaluated, representing additional potential sources of variation in the reference population. Although age related differences were not a primary focus of this study, clinically abnormal birds were older than clinically normal birds. Age-related effects on avian plasma protein electrophoretic fractions remain poorly characterized; therefore, the potential specific contribution of age to the electrophoretic variation observed in this study cannot be determined. Future studies including larger, age-stratified populations may help determine the influence of age on electrophoretic profiles and whether age-specific reference intervals are significant in avian medicine.

This study demonstrates key analytical and clinical differences between AGE and CZE in macaws and highlights the complementary diagnostic value of hematologic and electrophoretic evaluation in psittacine birds. The absence of significant electrophoretic differences between clinically normal and abnormal birds should be interpreted as an exploratory finding, with future studies involving larger, more homogenous sample populations necessary to better determine the clinical implications of these methods in diseased birds. Prospective longitudinal studies integrating advancing electrophoresis methods with avian acute-phase protein biomarkers may further clarify the clinical and research applications of these complementary diagnostic tools. Future studies specifically evaluating the differences between subspecies, age, sex, and reproductive status may further clarify the significance of suspected sources of electrophoretic variation in avian medicine and strengthen reference interval generation.

## Data Availability

The raw data supporting the conclusions of this article will be made available by the authors, without undue reservation.
